# Intergroup Meta-Respect Perceptions in a Context of Conflict

**DOI:** 10.3390/bs15111474

**Published:** 2025-10-29

**Authors:** Meytal Nasie

**Affiliations:** School of Education, Tel Aviv University, Tel Aviv 6997801, Israel; metalnas@tauex.tau.ac.il

**Keywords:** respect, meta-respect, intergroup meta-perceptions, intergroup conflict

## Abstract

Intergroup relations in contexts of conflict are often characterized by mutual disrespect. The present research introduces and examines the concept of intergroup meta-respect—the belief that one’s ingroup is viewed by the outgroup as deserving of respect. Across two studies conducted among Jewish and Arab citizens of Israel, we investigated perceptions of outgroup deservedness of respect, meta-respect, and their implications for intergroup attitudes. Study 1 (*N* = 451) revealed systematic biases in meta-respect: both groups underestimated the extent to which the outgroup considered their ingroup deserving of respect as human beings. Arabs, however, demonstrated greater accuracy and positivity, perceiving Jews as more deserving of respect than Jews perceived Arabs. Study 2 (*N* = 326) experimentally tested interventions aimed at correcting these misperceptions by presenting participants with accurate survey data, either with or without explicit correction of misperceptions. Exposure to corrective information increased participants’ feelings of respect, hope, and positive perceptions of the outgroup, and indirectly—through feelings of respect—enhanced willingness to respect and interact with the outgroup, although these effects were more limited among minority (Arab) participants. These findings highlight the critical role of meta-respect in shaping intergroup dynamics, and suggest that interventions targeting respect perceptions hold potential for improving intergroup relations, even amid ongoing intractable conflict.

## 1. Introduction

Relations between Jews and Arabs in Israel are deeply entrenched in the ongoing Israeli–Palestinian intractable conflict (Bar-Tal, 2013) and are marked by persistent tension (Reiter, 2023). These relations took a significant turn following Hamas’s attack in October 2023 and the subsequent war between Israel and Hamas. In the aftermath of the war’s outbreak, more than half of both Jewish and Arab citizens of Israel reported a worsening in intergroup relations (Deitch et al., 2023). During this period, members of both groups experienced an intensified sense of threat, driven by fears of the other side and reinforced by the misperception that the opposing group is inclined toward violence (Nassir et al., 2023).

A central feature of intractable conflicts is the delegitimization of the adversary (Bar-Tal & Hammack, 2012). This process frequently involves the denial of the outgroup’s humanity and may be reflected in perceptions that its members are undeserving of respect, as well as in the justification of behaviors that diminish or withhold respect from them (Nasie, 2016). Beyond direct perceptions, intergroup relations are also shaped by meta-perceptions—beliefs about how one’s own group is viewed by the outgroup (Moore-Berg, 2024; Vorauer et al., 1998; Yzerbyt et al., 2013). Meta-perceptions are frequently biased, inaccurate, and disproportionately negative (Lees & Cikara, 2020; Moore-Berg et al., 2020), particularly in contexts of conflictual relations (Nir et al., 2023). In the context of conflict, group members adopt socio-psychological mechanisms that help them cope with the threats and uncertainty generated by the conflict (Bar-Tal, 2007, 2013). These mechanisms serve to strengthen a positive social identity by sharpening the distinction between the ingroup and the outgroup (Tajfel & Turner, 1979). Consequently, the outgroup is often portrayed in a negative light, accompanied by feelings of aversion and bias, and is attributed with hostile traits and harmful intentions (Bar-Tal, 1998; Halperin, 2008; Stephan & Stephan, 2000).

The present research introduces and examines a novel extension of meta-perceptions concept: *intergroup meta-respect*. Intergroup meta-respect refers to individuals’ beliefs about whether members of an outgroup consider their ingroup deserving of respect as human beings. This construct opens a new avenue for examining how meta-perceptions of respect influence intergroup attitudes and may provide leverage points for interventions in conflict contexts.

### 1.1. Definition of Respect

Drawing on in-depth research among Israeli Jews and Palestinians, Nasie (2016, 2023b) proposed a multidimensional comprehensive definition of respect comprising four key dimensions: (1) Avoiding Disrespect—This dimension emphasizes maintaining respect by refraining from negative behaviors that could undermine it. It is framed as a set of prohibitions outlining what should not be done to preserve respect. (2) Deserved/Normative Respect—This dimension refers to appropriate and polite behavior rooted in external social norms, manners, and fundamental human rights that all individuals inherently deserve. It is framed as a set of positive obligations that dictate what ought to be done. (3) Conditional Respect—This form of respect is contingent on specific qualities, achievements, or actions. It includes two key aspects: recognition of accomplishments and traits, and the principle of reciprocity in mutual respect. (4) Considerate Respect—This dimension involves acknowledging and addressing the unique physical, social, and emotional needs of others, respecting their perspectives, differences, and existence.

For the purpose of the present research, only one dimension—deserved/normative respect—is used to define respect, as it encapsulates fundamental human dignity and socially accepted respectful behavior. This dimension reflects a basic, universally recognized mode of acknowledging others as human beings who inherently merit respect (see e.g., Stith, 2004). Moreover, three previous studies (Nasie, 2023a) consistently showed that the four identified dimensions of respect contribute similarly to perceptions of being respected. This consistent pattern offers empirical justification for focusing on a single dimension in the present research, thereby enhancing conceptual clarity while maintaining theoretical and empirical rigor.

### 1.2. Intergroup Respect

Intergroup respect has been studied across a variety of social and political contexts, consistently showing that when groups receive respectful treatment from outgroups, they report more positive emotions, develop more favorable intergroup attitudes, and display reduced biases. Research demonstrates that respectful treatment—particularly in the form of positive social evaluations—fosters emotions such as pride while diminishing negative emotions like shame (Ellemers et al., 2004).

Empirical work highlights how equality-based respect shapes intergroup attitudes. In Germany, when members of the gay and lesbian community felt respected by the Muslim community, their attitudes toward Muslims became less negative. Similarly, when they perceived respect from the German majority, their anti-Muslim sentiment declined (Simon & Grabow, 2014). Equality-based respect has also been linked to a greater willingness to recategorize ingroup and outgroup members into a shared identity (e.g., as Americans), a well-documented strategy for reducing ingroup favoritism and fostering positive outgroup evaluations (Simon et al., 2015).

Acar et al. (2024) extended this work in a large cross-national study spanning six countries (e.g., Turkish participants in relation to Kurds and Arabs, White Americans in relation to Black and Latinx groups, and Flemish participants in relation to ethnic minorities). Across advantaged and disadvantaged groups, they found that perceived outgroup respect served as a significant emotional mediator of positive direct and indirect contact effects, beyond typical mediators such as outgroup threat and trust, and these effects persisted over time.

The political domain likewise illustrates the significance of intergroup respect. Experimental research shows that inducing equality-based respect between students with opposing political views reduces bias against outgroup arguments (Eschert & Simon, 2019). In the context of intergroup conflict, Nasie (2023a) demonstrated that when Jewish Israelis perceived respect from Palestinians, their attitudes toward both the adversary group and the conflict improved. Moreover, respect appears to operate in a reciprocal cycle: expressions of respect from one party increase the likelihood of receiving respect in return (Nasie, 2016; Simon & Schaefer, 2018).

Research on intergroup respect emphasizes that respect functions both as recognition of individuals’ inherent worth and as a group-level norm affirming equality and inclusion (Simon & Grabow, 2014; Simon et al., 2015). Within this broader framework, recognition-based mechanisms refer to social-psychological processes through which individuals or groups seek acknowledgment of their moral worth, social identity, and legitimate status from others. Respect represents one such mechanism—one that specifically affirms the other’s humanity and equal standing. Other recognition-based processes, such as fairness, moral acknowledgment, or apology, may operate alongside or even in tension with respect. For example, a group may be treated fairly according to procedural norms yet still feel disrespected or excluded. Building on this framework, the present research examines intergroup meta-respect—the perception that one’s ingroup is respected by an outgroup—as a distinct form of recognition that captures perceived acknowledgment and validation from the outgroup.

### 1.3. Intergroup Meta-Perceptions

People expect to be perceived positively by their ingroup and negatively by outgroups (Frey & Tropp, 2006). Vorauer et al. (1998) conducted a series of studies that examined the existence of intergroup meta-stereotypes and their implications for intergroup attitudes and feelings toward intergroup interaction. Their first study indicated that White Canadians hold meta-stereotypes regarding how they are viewed by Aboriginal Canadians and that these meta-stereotypes included considerable number of negative attributes. Their second study showed that people expect an outgroup member to attribute to them typical stereotypes that exist toward their ingroup. The more participants expected to be stereotyped, the less they expected to enjoy contact with an outgroup member and the more they expected to experience negative emotions during the interaction.

Negative meta-perceptions may also lead to intergroup anxiety—anxiety that people experience when interacting or anticipating interacting with outgroup members (Stephan & Stephan, 1985). Finchilescu (2010) investigated the role of prejudice and meta-stereotypes in the experience of intergroup anxiety during contact simulation among students in South Africa. The participants believed that they were communicating in an Internet chatroom with two other students who were either of their same race (the intragroup contact condition) or of another race (the intergroup contact condition). They participated in an interactive task, and then they were asked to rate their feelings about their participation. In the intergroup condition, the level of prejudice and the degree of meta-stereotypes were both found to be predictors of intergroup anxiety, and meta-stereotypes were found to be predictors of intergroup anxiety at a higher level.

In contrast, positive meta-perceptions, which are the focus of the current research, have the potential to foster more positive intergroup relations (Vezzali, 2017). Participants in Vezzali’s (2017) study were Italian high school students who were told they were going to meet African immigrants. The researcher manipulated the meta-stereotype valence, presenting positive vs. negative traits that African immigrants allegedly assigned to Italians. The results showed that the activation of positive meta-stereotypes led the Italian students to anticipate greater enjoyment of an upcoming interaction with African immigrants by increasing positive feelings about the future contact. In Kteily et al.’s (2016) study, American participants who learned that Muslims humanize Americans (meta-humanization through Ascent Dehumanization scale) humanized Muslims in turn. Research on meta-humanization in conflict contexts (Kosovo and North Macedonia) has demonstrated that learning the outgroup views one’s ingroup as human (in terms of being evolved and civilized) enhances both willingness to accept help and engage in intergroup contact, mediated by more positive attributions to outgroup actors (Borinca et al., 2021). Stathi et al. (2020) examined the role of contact meta-perceptions on positive intergroup contact and outgroup attitudes in three contexts: international students’ view of British students, general public views of people with schizophrenia, and both Muslims’ and non-Muslims’ views of one another. Among these three intergroup relationships, the perception of the outgroup’s desire for intergroup contact was consistently highlighted as predictor of intergroup contact which in turn predicted positive outgroup attitudes.

The present research builds on this prior work on positive meta-perceptions. Each of these studies highlights the importance of perceiving that the outgroup views one’s ingroup positively, yet they focus on different dimensions of intergroup perception. *Meta-respect* emphasizes perceived moral recognition and deserved treatment, complementing the cognitive (meta-stereotype and meta-humanization) and relational (contact meta-perceptions) dimensions documented in earlier studies.

### 1.4. The Accuracy or Inaccuracy of Meta-Perceptions

Meta-perceptions in intergroup contexts are often inaccurate, as groups tend to overestimate the negativity of outgroup attitudes and intentions toward them (Frey & Tropp, 2006; Moore-Berg et al., 2020; Vorauer et al., 2000). That highlights the need for interventions to correct these misperceptions and improve intergroup relations (Moore-Berg & Hameiri, 2024). This approach draws on inconsistency theories, which emphasize the aversive psychological state that arises when individuals encounter information that contradicts their existing beliefs or expectations (e.g., cognitive dissonance theory, Festinger, 1957; see also Cooper & Fazio, 1984; McGrath, 2017). To reduce this discomfort and restore a sense of coherence, individuals are often motivated to adjust their prior attitudes or beliefs (Bar-Tal & Hameiri, 2020). Indeed, interventions aimed at correcting meta-perceptions have been shown to be effective in reducing biases and fostering more constructive intergroup dynamics (for review see Moore-Berg, 2024). For example, Nir et al. (2023) conducted a series of studies among Jews and Arabs in the context of the Israeli-Palestinian conflict. The first study revealed that although the vast majority of Jews and Arabs opposed violence, each group perceived their respective outgroup as significantly less opposed to such violence. The second study found that experimentally exposing Jewish and Arab citizens to corrective information showing that their outgroup vastly opposes violence increased participants’ own opposition to intergroup violence. A third study, conducted during an escalation in the conflict, replicated these results, while demonstrating that the intervention was successful in both increasing opposition to violence and decreasing support for violence.

### 1.5. The Present Research

The present research examines meta-respect perceptions of two groups positioned on opposing sides of a conflict, as well as their impact on intergroup attitudes. The research comprises two studies. In Study 1, I aim to examine intergroup respect perceptions, including meta-perceptions—that is, how individuals believe members of their own group are perceived in terms of respect by the outgroup. I will further assess the accuracy of these meta-perceptions and explore their relationship with the tendency to perceive the outgroup as human. Study 1 will generate empirical data on respect perceptions and meta-perceptions among both Jews and Arabs, providing an accurate picture of how each group views and believes it is viewed by the other. In Study 2, I aim to test the effects of an intervention in which participants are presented with the “true” survey data of Study 1—either correcting their meta-respect perceptions or exposing them to the outgroup’s positive respect attitudes without directly correcting their meta-perceptions. Although both intervention types convey positive outgroup regard, they may activate distinct psychological processes. Corrective meta-respect messages communicate that the outgroup holds more respect for the ingroup than previously assumed, thereby confronting an existing belief discrepancy. This corrective information challenges entrenched expectations of disrespect or devaluation and may induce a state of cognitive inconsistency that motivates individuals to adjust their attitudes to restore coherence. In contrast, direct expressions of respect do not challenge prior expectations but rather affirm the ingroup’s moral worth, engaging affirmation processes without invoking dissonance reduction processes. It is also important to consider that the psychological meaning of meta-respect may vary across group status positions. For members of low-status or historically disadvantaged groups (Arabs citizens of Israel), perceiving that the high-status majority (Jews in Israel) accords them deserved or normative respect serves as acknowledgment of their moral worth and social legitimacy, thereby counteracting chronic experiences of perceived marginalization and exclusion. In contrast, for members of high-status majority groups, perceiving respect from a lower-status minority may primarily affirm their moral self-image and sense of fairness rather than challenge their social legitimacy. These status-based asymmetries suggest that meta-respect may fulfill distinct psychological functions across groups and thus produce divergent reactions (see in this context, Nasie, 2016; Shnabel & Nadler, 2008).

This design enables examination of whether correcting meta-perceptions of respect, or simply highlighting outgroup positivity, can foster more favorable intergroup attitudes. These outcomes include perceiving the outgroup as human, experiencing more positive feelings and perceptions toward them (e.g., hope, seeing them in positive light), recognizing their worthiness of respect, and expressing greater willingness for intergroup contact.

Based on the above literature review, the hypotheses of the present research are as follows:

1. Jewish and Arab participants will inaccurately perceive their respective outgroups as significantly less respectful toward them than the outgroups actually report, reflecting an outgroup-negativity bias in meta-perceptions.
2. Higher levels of respect toward the outgroup, as well as higher levels of perceived outgroup respect (meta-respect), will be positively associated with perceiving the outgroup as human.
3. Presenting participants with information that directly challenges misperceptions—specifically, evidence that the outgroup expresses higher levels of respect than assumed—will foster more positive intergroup attitudes. These improvements are expected to manifest in heightened positive feelings and perceptions toward the outgroup, greater acknowledgment of its worthiness of respect, and stronger willingness for intergroup contact.
4. Presenting evidence of outgroup respect without explicitly correcting misperceptions will also promote positive attitudes, although the magnitude of its effect relative to the corrective intervention remains an open empirical question.

## 2. Study 1

### 2.1. Method

#### 2.1.1. Participants

Study 1 included 451 Jewish and Arab participants. The Jewish sample was representative and consisted of 252 adult Israeli Jews: 52% women and 48% men (*M*_age_ = 33.65, *SD*_age_ = 9.46). In terms of political orientation, approximately 71% of these participants defined themselves as between being moderately to extremely rightist, approximately 20% as centrist, and approximately 9% as moderately to extremely leftist. In terms of religiosity, approximately 45% defined themselves as secular, approximately 37% as traditional, approximately 9% as religious and approximately 9% as ultra-orthodox.

The Arab sample consisted of 199 adult Arab citizens of Israel: 61% women and 39% men (*M*_age_ = 35.25, *SD*_age_ = 7.96), 68% Muslims, 14% Christian, and 18% Druze. In terms of religiosity, approximately 36% defined themselves as secular, approximately 46% as traditional, and approximately 18% as religious. In terms of political orientation, approximately 33% of these participants voted in the last elections in 2022 for Arab parties, approximately 11% voted to Jewish parties, approximately 37% did not vote, and approximately 19% did not identify their choice.

Both Jewish and Arab samples recruited during February 2025 from the general adult Jewish and Arab population in Israel using an online survey platform (‘Panel4all’) that offers monetary compensation in return for participation in surveys. The sample size in all studies was determined a priori using G*Power version 3.1.9.7 (Cohen’s *d* medium effect size 0.50 for Study 1 and Cohen’s *f* medium effect size 0.25 for Study 2, 90% power, *α* = 0.05). The research was approved by the Institutional Review Board. Protocol no. 0004286-3. The data that support the findings of this research are available in OSF at: https://osf.io/4zejk/overview?view_only=79939bea7ecb4b778ee5fae9d892d6d8 (accessed on 24 October 2025). 

#### 2.1.2. Measures

*Outgroup deservedness of respect* was assessed with a single item. Participants were asked: “*To what extent do you think that the outgroup (Arabs/Jews, respectively) deserves respect as human beings?*”, Responses were given on a scale from 1 (totally undeserving of respect) to 6 (strongly deserving of respect).

*Meta-respect toward the outgroup* was assessed with a single item. Participants were asked: “*To what extent do you think that Arabs/Jews believe that Jews/Arabs deserve respect as human beings?*” Responses were given on the same 1 (totally undeserving of respect) to 6 (strongly deserving of respect) scale.

*Outgroup humanization* was measured with a single item adapted from McDonald et al. (2017). Participants were asked: “*To what extent do you see Arabs/Jews as human?*” Responses were given on a scale from 1 (not at all) to 10 (to a great extent).

*Political orientation for Jewish participants* was measured with a single item: “*How would you describe your political orientation?*” Responses were given on a five-point scale: 1 = extreme right, 2 = right, 3 = center, 4 = left, and 5 = extreme left.

*Political orientation among Arab participants* was measured with a single item. Participants were asked to indicate the party they voted for in the 2022 elections, using a six-point scale: 1 = Hadash-Ta’al (Arab party), 2 = Ra’am (Arab party), 3 = Balad (Arab party), 4 = Zionist parties, 5 = did not vote, and 6 = other.

#### 2.1.3. Procedure

Participants were invited to take part in a study designed to examine social attitudes. After providing informed consent, they responded to a series of questions assessing their perceptions of the outgroup’s deservedness of respect, their meta-respect perceptions, their perceptions of outgroup humanization, and their political orientation. As part of the broader research project, additional exploratory measures were included; these are not the focus of the present report.

### 2.2. Results

#### 2.2.1. Outgroup Deservedness of Respect

As presented in the frequency tables (Table 1 and Table 2, left columns), about 64% of Jewish participants and about 84% of the Arab participants stated that they believed the outgroup deserved respect as human beings. A comparison between the groups revealed that Arabs (*M* = 4.88, *SD* = 1.27) perceived Jews as deserving respect significantly more than Jews (*M* = 3.96, *SD* = 1.75) perceived Arabs as deserving respect, *t*(446.11) = −6.45, *p* < 0.001, *d* = −0.59.

#### 2.2.2. Meta-Respect Toward the Outgroup

As presented in the frequency tables (Table 1 and Table 2, right columns), only about 30% of Jewish participants and about 46% of the Arab participants stated that they believed the outgroup regarded their ingroup as deserving respect as human beings.

#### 2.2.3. Accuracy/Inaccuracy of Meta-Respect

**Figure 1:**
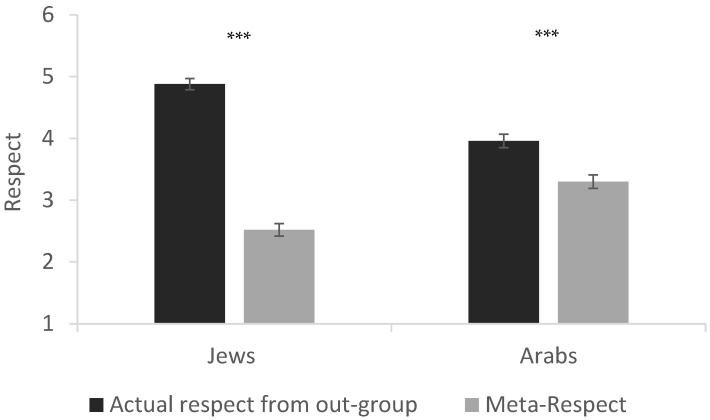
Actual Respect and Meta-Respect. Note. *** *p* < 0.001. Error bars represent standard errors. N_Jews_ = 252, N_Arabs_ = 199.

To examine the accuracy/inaccuracy of intergroup meta-respect, we compared meta-respect inferred by each group to the actual level of respect expressed by the respective outgroup. Overall, both groups *underestimated* the extent to which the outgroup respects their ingroup; however, this gap was more pronounced among Jewish participants: Jews’ meta-respect versus Arabs’ actual respect (*M_difference_* = 2.36; *t*(251) = 22.88, *p* < 0.001, CI [2.15, 2.56], *d* = 1.44); Arabs’ meta-respect versus Jews’ actual respect (*M_difference_* = 0.66; *t*(198) = 5.87, *p* < 0.001, CI [0.44, 0.88], *d* = 0.41). In other words, the magnitude of the meta-perception negativity bias—reflected in the gap between actual outgroup respect and perceived outgroup respect (meta-respect)—was greater among Jews than among Arabs, *t*(449) = 11.05, *p* < 0.001, CI [1.39, 1.99], *d* = 1.04 (see Figure 1).

#### 2.2.4. Correlations Among Outgroup Deservedness of Respect, Meta-Respect, and Outgroup Humanization

Table 3 and Table 4 show that perception of outgroup deservedness of respect are highly correlated with perceptions of outgroup humanization and moderately correlated with meta-respect. In addition, meta-respect perceptions are moderately correlated with outgroup humanization. This means that the more participants perceived the outgroup as deserving respect, the more they also tended to perceive the outgroup as human and to believe that the outgroup respected their ingroup

### 2.3. Discussion

Study 1 revealed that the meta-perceptions regarding respect held by both Arabs and Jews are generally inaccurate and negatively biased. That is, both groups underestimated the extent to which the outgroup respects their ingroup. These findings are consistent with previous research showing that meta-perceptions toward outgroups tend to be inaccurate and are often negatively biased—both in contexts involving non-conflictual groups and those involving groups in conflict (Frey & Tropp, 2006; Lees & Cikara, 2020; Moore-Berg et al., 2020; Nir et al., 2023; Yzerbyt et al., 2013). Despite the overall inaccuracy, Arab participants were relatively more accurate in their meta-respect perceptions compared to Jewish participants. In addition, Arabs expressed more positive views than Jews regarding the outgroup’s deservingness of respect and their perception of the outgroup as human. These perceptual gaps between Jews and Arabs will be further elaborated and discussed in the General Discussion, particularly in relation to their respective positions as minority and majority groups within the Israeli social context.

Finally, the findings indicate that meta-respect is linked to the extent to which individuals perceive the outgroup as deserving of respect. This association may suggest the existence of a reciprocal cycle: perceptions of being respected by the outgroup (meta-respect) foster a greater tendency to perceive the outgroup itself as deserving of respect. Such a dynamic highlights the importance of enhancing positive meta-respect perceptions, as they may serve as a psychological gateway to promoting mutual recognition and more respectful intergroup relations.

In Study 2, I aim to examine the effects of presenting Jewish and Arab participants with the actual survey data of Study 1—thereby correcting their meta-respect perceptions—compared to exposing them to the outgroup’s actual positive respect attitudes without directly correcting their misperceptions. This design draws on prior interventions, including one that corrected meta-perceptions about violence in the context of conflict (Nir et al., 2023, discussed in detail above), as well as another that framed outgroup statements as expressions of respect (Nasie, 2023a). In the latter intervention, Israeli Jewish participants were presented with respect expressions allegedly made by Palestinians in the form of online comments or posts. These expressions were perceived as respect from the adversary group, and this perceived respect, in turn, predicted improved attitudes and perceptions toward both the adversary group and the conflict. The present study builds on and extends these approaches by directly targeting the domain of *respect* through the use of real survey data from both sides of the conflict. In doing so, it allows for a unique test of whether correcting misperceptions or merely activating genuine outgroup respect can serve as effective pathways for fostering more positive intergroup attitudes.

## 3. Study 2

### 3.1. Method

#### 3.1.1. Participants

Study 2 included 326 Jewish and Arab participants. The Jewish sample was representative and consisted of 165 adult Israeli Jews: 53% women and 47% men (*M*_age_ = 33.09, *SD*_age_ = 9.12). In terms of political orientation, approximately 67% of these participants defined themselves as between being moderately to extremely rightist, approximately 24% as centrist, and approximately 9% as moderately leftist. In terms of religiosity, approximately 46% defined themselves as secular, approximately 36% as traditional, approximately 10% as religious and approximately 8% as ultra-orthodox.

The Arab sample consisted of 161 adult Arab citizens of Israel: 60% women and 40% men (*M*_age_ = 34.18, *SD*_age_ = 8.33), 70% Muslims, 12% Christian, and 18% Druze. In terms of religiosity, approximately 35% defined themselves as secular, approximately 44% as traditional, and approximately 21% as religious. In terms of political orientation, approximately 33% of these participants voted in the last elections in 2022 for Arab parties, approximately 14% voted to Jewish parties, approximately 37% did not vote, and approximately 16% did not identify their choice.

Both Jewish and Arab samples recruited during February and March 2025 from the same online survey platform as Study 1. None of the Jewish participants in Study 2 had participated in Study 1. However, most of the Arab participants in Study 2 (81%) had also participated in Study 1, due to the limited availability of Arab respondents in the online survey company’s panel. It should be noted that although the two studies shared several core measures (e.g., respect and meta-respect), their procedures were distinct: Study 1 was purely correlational, whereas Study 2 introduced an experimental manipulation and additional outcome variables. Importantly, the time interval between studies—Study 1 conducted between 12–21 February 2025, and Study 2 between 25 February–9 March 2025—reduces the likelihood of systematic carryover or memory effects. Given these considerations, and because the designs served distinct purposes and the dependent measures in Study 2 were assessed anew under different experimental conditions, any residual dependence between repeated participants is unlikely to have biased the observed effects.

#### 3.1.2. Measures

All measures were rated on a 5-point Likert scale ranging from 1 (not at all) to 5 (to a great extent), unless otherwise specified.

*Meta-respect toward the outgroup* was measured with two items. The first item was identical to that used in Study 1. The second item asked participants: “*In your opinion, what percentage of Arabs/Jews believe that Jews/Arabs deserve respect as human beings?*” Responses were given on a scale ranging from 0% (no Arabs/Jews think that Jews/Arabs deserve respect) to 100% (all Arabs/Jews think that Jews/Arabs deserve respect).

*Feeling of respect* was measured with a single item: “*To what extent did the attitudes of Arabs/Jews in the text you read make you feel respected?*”

Hope regarding intergroup relations was measured with a single item adapted from Cohen-Chen et al. (2015): “*To what extent did the attitudes of Arabs/Jews in the text you read make you feel hope regarding future relations between Arabs and Jews?*”

*Seeing the outgroup* in a positive light was measured using a two-item scale adapted from Nasie (2023a): “*To what extent did the attitudes of Arabs/Jews in the text you read make you think about Arabs/Jews in a more positive light than before?*” “*make you think that Arabs/Jews have positive aspects*”? (α = 0.78 for Jews, α = 0.79 for Arabs).

*Outgroup humanization* was measured as in Study 1.

*Willingness to respect the outgroup* was measured using a three-item scale adapted from Nasie (2023a). Participants were asked to indicate to what extent they were “*willing to express respect to Arabs/Jews*”, “*think the Arabs/Jews deserved respectful regard*” and “*think Jews/Arabs should respect the Arabs/Jews*” (α = 0.93 for Jews, α = 0.90 for Arabs).

*Willingness to interact with the outgroup* was measured using a four-item scale adapted from Voca et al. (2022). Participants were asked to indicate to what extent they agreed or not with the items: “*I would be happy if I had an opportunity to engage in contact with Jews/Arabs*,” “*Working together with Jews/Arabs would be no problem for me,*” “*I would feel okay if some of my direct neighbours were Jews/Arabs*,” and “*I would be happy to personally get to know more Jews/Arabs*”. Scores ranged from 1 (*do not agree at all*) to 7 (*strongly agree*), with higher scores indicating greater willingness to interact. The measure demonstrated high reliability (α = 0.91 for Jews; α = 0.90 for Arabs).

*Political Orientation for Jews and Arabs* was measured as in Study 1.

#### 3.1.3. Procedure

Participants were invited to take part in a study on social attitudes. After providing informed consent, they were randomly assigned to three conditions—outgroup respect, meta-respect correction, and control. All participants first answered the two meta-respect questions and then read one of the following three texts, corresponding to their assigned condition. These texts represented the two experimental manipulations (out-group respect and meta-respect correction) and a control condition, respectively, designed to test the effects of perceived respect from the out-group and the correction of meta-respect. To ensure ecological validity, the texts were tailored separately for Jewish and Arab participants based on the real-world results of Study 1. The texts were presented as descriptions of survey results.

*The texts for Jewish participants:* (a) “a research conducted recently among Arabs in Israel revealed that 84% of the Arabs, who constitute the majority of the Arabs in the survey, said that Jews deserved respect as human beings” (*n* = 55). (b) “a research conducted recently among Jews and Arabs in Israel revealed that only 30% from the Jews said that Arabs think that Jews deserved respect as human beings. However, actually, 84% of the Arabs, who constitute the majority of the Arabs in the survey, said that Jews deserved respect as human beings” (*n* = 56). (c) “a research conducted recently among Arabs in Israel revealed that 84% of the Arabs, who constitute the majority of the Arabs in the survey, said that they like to eat rice” (*n* = 54).

*The texts for Arab participants:* (a) “a research conducted recently among Jews in Israel revealed that 64% of the Jews, who constitute the majority of the Jews in the survey, said that Arabs deserved respect as human beings” (*n* = 55). (b) “a research conducted recently among Arabs and Jews in Israel revealed that only 46% of the Arabs said that Jews think that Arabs deserved respect as human beings. However, actually, 64% from the Jews, who constitute the majority of the Jews in the survey, said that Arabs deserved respect as human beings” (*n* = 53). (c) “a research conducted recently among Jews in Israel revealed that 64% of the Jews, who constitute the majority of the Jews in the survey, said that they like to eat rice” (*n* = 53).

After reading the text, the participants were required to answer an attention verification question correctly. Participants who failed to answer this question (*n*_Jews_ = 17, *n*_Arabs_ = 28) were excluded from the sample, and new participants were sampled in their place. The demographic profile of excluded participants did not differ substantially from the full sample (see Supplementary Table S1). The attention question was followed by measures of the above dependent variables.

### 3.2. Results

#### 3.2.1. Meta-Respect Toward the Outgroup

First, I calculated the baseline level of meta-respect prior to the intervention (see Table 5 and Table 6). The findings replicate those of Study 1, indicating that only about 21% or 30% (depending on the specific question) of Jewish participants and about 48% or 46% of Arab participants believed that the outgroup perceived their ingroup as deserving respect as human beings. These results mirror the patterns observed in Study 1 and further validate them across two different measures of meta-perception. It should be noted that testing the difference between the two metrics—after rescaling the 0–100% meta-estimate to a 1–6 scale—revealed no significant difference between them in both samples. Jewish sample: *M*_1–6 scale_ = 2.35, *SD* = 1.32; *M*_1–6 rescaled_ = 2.47, *SD* = 1.43, *t*(164) = −1.18, *p* = 0.23, CI [−0.33, 0.08], *d* = −0.09. Arab sample: *M*_1–6 scale_ = 3.34, *SD* = 1.47; *M*_1–6 rescaled_ = 3.30, *SD* = 1.43, *t*(160) = 2.84, *p* = 0.77, CI [−0.20, 0.27] *d* = 0.02. These results indicate that participants’ meta-perceptions were highly consistent across response metrics.

#### 3.2.2. The Effect of Outgroup Respect and Meta-Respect Correction on Intergroup Attitudes

The main analyses examined the effects of outgroup respect and meta-respect correction on participants’ attitudes. A one-way ANOVA was conducted with condition as the independent variable and feelings and attitudes as the dependent variables. The results, presented in Table 7 and Table 8, indicate that in both samples there was a significant effect of the experimental conditions—outgroup respect and meta-respect correction—compared to the control condition (and with no difference between the two experiment conditions) on feelings of respect, hope, and perceptions of the outgroup in a positive light. In the Arab sample, there was also a significant effect on perceived outgroup humanization, but this emerged only in the outgroup-respect condition.

No significant effects of the outgroup respect or meta-respect correction conditions were found in either sample on willingness to respect the outgroup or to engage in intergroup contact. In addition, among Jewish participants, no effect emerged on perceived outgroup humanization. Prior research suggests that part of the association between adversary group respect expressions and positive intergroup attitudes is indirect, operating through *perceived respect* (Nasie, 2023a). Accordingly, in the following analysis I test the indirect effects of the experimental conditions on willingness to respect the outgroup and to interact with its members (and, for the Jewish sample, on outgroup humanization) through *feelings of respect*. This approach is grounded in the notion that respect is inherently subjective: for a gesture or expression to have an impact, it must be perceived as respectful by the recipient. Thus, only participants who genuinely experience respect in response to the adversary’s expression are likely to alter their intergroup attitudes (Nasie, 2023a).

Therefore, a mediation model was tested with experimental condition as the predictor, feelings of respect as the mediator, and intergroup outcomes as the dependent variables. Hayes’ PROCESS macro for SPSS (version 4.2, Model 4) was used with 5000 bootstrap samples for each dependent variable. Table 9 presents the standardized significant indirect effects of each condition (outgroup respect vs. control; meta-respect correction vs. control) on intergroup attitudes through feelings of respect in the Jewish sample, controlling for political orientation (analyses without control yielded similar results).

As shown in Table 9, the experiment conditions produced a significant positive indirect effect on intergroup attitudes via feeling respect from adversary group members. Specifically, the more Jewish participants felt respected by Arabs through the text they read, the more they perceived the outgroup as human, reported greater willingness to respect them, and indicated higher willingness for intergroup contact (the latter effect was significant only in the outgroup respect condition). In contrast, no significant indirect effects were observed in the Arab sample; therefore, these results are not presented in detail.

### 3.3. Discussion

The findings of Study 2 provide partial support for the hypotheses regarding the effects of outgroup respect and meta-respect correction on intergroup attitudes. Consistent with expectations, exposure to outgroup respect and to corrective information about meta-respect led to improvements in participants’ feelings of respect, hope, and overall positive perceptions of the outgroup, relative to the control condition. Notably, in the Arab sample, the outgroup-respect condition also enhanced perceptions of outgroup humanization, whereas this effect did not emerge in the meta-respect correction condition.

Importantly, however, the interventions did not directly influence willingness to respect the outgroup, willingness for intergroup contact, or, in the Jewish sample, perceptions of outgroup humanization. This aligns with prior evidence suggesting that the impact of adversary-group expressions of respect on attitudes is often indirect and mediated through the subjective perception of respect (Nasie, 2023a). Mediation analyses confirmed this pattern: in the Jewish sample, the outgroup respect and the meta-respect correction conditions produced significant indirect effects on multiple intergroup outcomes through feelings of respect. In other words, the more participants in both conditions felt genuinely respected by Arabs, the more they perceived the outgroup as human and expressed greater openness to mutual respect and contact. These findings underscore the central role of feeling respect in shaping intergroup attitudes. By contrast, no significant indirect effects were observed among Arab participants, which may, in part, reflect their minority status and different expectations regarding respect within the conflict context as will be discussed in the General Discussion section.

It should be acknowledged that some of the outcome variables were measured using single-item scales. This approach was chosen to simplify the measurement procedure and directly capture the specific outcomes of interest. However, it limits the ability to assess measurement reliability and may only partially reflect the complexity of respect-related emotions and attitudes. Future research should employ multi-item scales to enhance construct validity and provide a more comprehensive assessment of these processes.

## 4. General Discussion

Intergroup relations in contexts of protracted conflict are often characterized by mutual displays of disrespect and delegitimization. Nevertheless, respect—as a universal value to which every individual is entitled by virtue of their humanity—may also be recognized even across adversarial group lines. That is, members of rival groups may still hold perceptions that outgroup members are deserving of respect simply because they are human beings. The present research provides evidence that intergroup respect perceptions do emerge even within the deeply divided context of Jewish–Arab relations in Israel. However, alongside perceptions of respect toward the outgroup, the findings also reveal the existence of meta-perceptions suggesting that most outgroup members do not consider the ingroup as deserving of respect as human beings. These findings align with the theoretical framework that conceptualizes intergroup meta-respect as a distinct form of recognition reflecting perceived acknowledgment and validation from the outgroup. They underscore that feeling recognized—or unrecognized—by the outgroup remains a central psychological dynamic within enduring conflict.

Across both studies, the findings demonstrated that Jews and Arabs hold inaccurate and negatively biased meta-respect perceptions, underestimating the extent to which the outgroup respects their ingroup. These findings are consistent with previous work showing that meta-perceptions are often systematically distorted in the direction of negativity (Frey & Tropp, 2006; Lees & Cikara, 2020; Moore-Berg et al., 2020; Nir et al., 2023; Yzerbyt et al., 2013). Such inaccurate meta-perceptions can have profound implications for the quality of intergroup relations (Lees & Cikara, 2020), as they distort social reality and lead individuals to underestimate the extent of positive attitudes held by the other side. Thus, the potential of outgroup respect to foster more positive intergroup attitudes (e.g., Nasie, 2023a; Simon & Grabow, 2014) is undermined.

Notably, Arab participants were relatively more accurate in their meta-respect perceptions than Jewish participants and expressed more positive views of the outgroup in terms of deservingness of respect and humanization. These asymmetries likely reflect the structural positions of the groups, with Jews occupying the majority and higher-status role, and Arabs representing a minority with lower status in the sociopolitical context in Israel (Bar-Haim & Semyonov, 2015). As prior literature suggests that minority groups often have more accurate and less negatively biased perceptions of majority groups, perhaps due to greater intergroup contact, attentional vigilance, or dependence on majority behaviors (e.g., Bettencourt et al., 2001; Tropp & Pettigrew, 2005). This asymmetry is consistent with broader evidence that members of disadvantaged groups are often more attentive to the perspectives of advantaged groups. Because minorities’ opportunities, safety, and social mobility depend heavily on the majority, they are motivated to carefully monitor majority attitudes and behaviors. Daily experiences of perceived discrimination and threat further increase minorities’ vigilance in detecting majority perceptions (Fiske & Dépret, 1996; Tropp & Pettigrew, 2005). Moreover, minorities are typically more immersed in majority’s language, culture, and institutions, which gives them greater familiarity with majority beliefs and norms. Their frequent intergroup interactions with majority members further reduce the likelihood of activating biased meta-perceptions, as they possess richer experiential knowledge of how the majority views them (Vorauer & Kumhyr, 2001). Together, these structural and psychological factors foster perspective-taking and more accurate inferences about how the majority views them (Saguy et al., 2008). Importantly, minorities may also be more positive in perceiving the outgroup as human and deserving of respect. As members of a lower-status group, affirming the humanity of the dominant majority can serve as a strategy to reduce conflict and maintain social harmony, while also reflecting an awareness of interdependence with the majority group (Saguy et al., 2008).

In contrast, members of the majority, whose higher status and security are less contingent on minority evaluations, often lack the same motivation to extend recognition or respect across group lines, resulting in both more biased meta-perceptions and less willingness to attribute respect to the minority outgroup. Moreover, the strongest biases in Jews’ meta-respect may also be understood in light of the extreme context of the ongoing war. Following the Hamas unprecedented attack of October 7, many Jews may find it difficult to believe that Arabs, as a collective, regard Jews as deserving of respect as human beings. In such a heightened context of fear and hostility, mistrust is intensified, and attributions of respect from the outgroup become even less plausible.

Study 2 extended these findings by testing whether interventions based on outgroup respect and meta-respect correction could improve intergroup attitudes. Consistent with predictions, exposure to both forms of intervention enhanced participants’ feelings of respect, hope, and positive perceptions of the outgroup, compared to the control condition, with no significant differences between the two intervention types. Notably, in the Arab sample, outgroup respect also increased perceptions of outgroup humanization, whereas this effect did not emerge in the meta-respect correction condition. At the same time, neither intervention directly influenced willingness to respect the outgroup, willingness for intergroup contact, or (in the Jewish sample) humanization. Instead, mediation analyses revealed that among Jews, the effect of outgroup respect on attitudes was indirect, operating through feeling respect. In other words, only when participants genuinely felt respected by Arabs did they update their intergroup perceptions, reporting greater humanization, willingness to respect, and willingness to engage in contact. These findings resonate with Borinca et al.’s (2021) evidence that the positive effects of intergroup meta-humanization on willingness to accept help and engage in contact are mediated by trust and positive attributions toward outgroup members. This suggests that while both meta-humanization and meta-respect may promote intergroup attitudes, they likely do so through different psychological mechanisms.

No indirect effects were observed among Arab participants, which may again reflect their minority status and distinct expectations regarding respect in the context of conflict. Arabs’ awareness of their lower social, economic, and political standing, alongside experiences of marginalization (Bar-Haim & Semyonov, 2015), likely shape these perceptions. More broadly, this finding is consistent with research showing that minority or low-status groups often exhibit heightened mistrust toward majority group members as a protective mechanism against perceived threats and unequal treatment (Dovidio et al., 2008; Lount & Pettit, 2012; Navarro-Carrillo et al., 2018). The finding that the intervention did not increase Arabs’ willingness to treat the out-group with respect or to interact with it suggests that respect-based corrections alone may be insufficient for minority populations to alter these outcomes.

Beyond status-based interpretations, alternative explanations should also be considered for the absence of the predicted indirect effects among Arab participants. Differences in the perceived credibility or personal relevance of the respect-related messages, as well as varying baseline expectations about outgroup respect, may have influenced participants’ responsiveness to the interventions. These factors could determine whether corrective or affirmational messages are perceived as genuine, credible, or personally meaningful. More broadly, the null findings may indicate boundary conditions of meta-respect interventions within minority group contexts, consistent with recent evidence that the positive effects of meta-perceptions on intergroup attitudes are not unconditional and may depend on the specific type of intergroup outcome assessed. For example, Borinca et al. (2024) found that while meta-humanization can reduce blatant dehumanization and foster openness to intergroup contact, it does not necessarily increase support for formal intergroup negotiation. Recognizing such boundary conditions helps situate meta-respect within a broader understanding of when positive meta-perceptions promote reconciliation and when their influence may encounter psychological or political limits. It is also important to acknowledge that the intervention stimuli were not entirely equivalent across groups, which may have influenced the observed asymmetry in effects. Specifically, Jewish participants were informed that a large majority of Arabs (84%) believed their group deserved respect, whereas Arab participants were told that a smaller proportion of Jews (64%) held this view. This asymmetry derived from the fact that the stimuli were based on real survey data rather than artificially balanced information. However, the resulting difference in message strength may have contributed to the weaker effects observed among Arab participants, beyond their minority status or broader status-based dynamics. The lower level of expressed respect in the message directed at Arab participants may have appeared less convincing or reassuring, thereby limiting its psychological impact.

The absence of significant effects on behavioral-intention outcomes, such as willingness to respect or interact may also reflect the nature of the underlying psychological mechanism targeted by the intervention. Because meta-respect operates through a sense of being respected—an internalized feeling of dignity, recognition, and moral worth—it may primarily influence self-relevant emotions and perceptions rather than directly motivating behavioral intentions. Experiencing respect can strengthen one’s sense of value and legitimacy without necessarily translating into a readiness for contact, particularly under conditions of active conflict. During periods of heightened tension and ongoing violence, such as the time in which the present study was conducted, behavioral engagement with the outgroup may be constrained by fear, security concerns, or social pressure, regardless of changes in perceived respect. Thus, meta-respect may function mainly as an internal recognition process rather than as an immediate driver of intergroup behavior. Future research should examine whether, and under what conditions, experiences of respect can extend beyond internal affirmation to influence behavioral intentions and actions.

Finally, it is important to interpret the findings within the broader sociopolitical context in which the study was conducted—during the aftermath of the October 7th events. In such period of acute conflict, collective trauma, fear, and anger profoundly shape intergroup perceptions and may constrain the potential for brief interventions to foster positive attitudes. Under these circumstances, shifts in hope or respect perceptions may represent modest but meaningful reductions in negativity rather than clear increases in positivity. The asymmetrical effects observed between Jewish and Arab participants may therefore reflect not only differences in group status but also distinct existential concerns and threat perceptions experienced by each community. Acknowledging these contextual realities underscores that the psychological space for mutual recognition and respect is sharply constrained during times of active conflict, highlighting both the limitations and the relevance of examining meta-respect processes under such extreme conditions.

In sum, the present research contributes to a growing body of work on meta-perceptions in conflict by documenting systematic biases in *meta-respect perceptions* and by testing interventions aimed at correcting them. The findings suggest that even within the context of intractable conflict, respect perceptions can still hold psychological significance, and that activating or correcting such perceptions may influence intergroup attitudes under certain conditions. Beyond their theoretical contribution, the findings also carry practical implications. They suggest that interventions grounded in respect can be useful in contexts of protracted conflict. Presenting members of adversary groups with credible evidence of mutual respect—or directly activating respect through carefully framed messages—can foster more positive attitudes. Such interventions may also be more acceptable than those focusing on sensitive issues such as violence, as respect represents a fundamental and universally valued dimension of human relations. The fact that these interventions relied on exposing participants to real data further enhances both their credibility and their potential for real-world application. At the same time, the asymmetries between Jews and Arabs underscore that group position shapes how such interventions are received. Efforts to design respect-based interventions must therefore take into account minority–majority dynamics.

Nevertheless, further research is needed to extend the validity of the current findings to other contexts of intergroup conflict. Testing these processes in conflicts that differ in intensity, asymmetry, and historical background would provide a broader understanding of when and how respect can serve as a constructive force in intergroup relations. In addition, future studies should examine how contextual and individual factors—such as prior intergroup contact experiences and exposure to conflict-related trauma—shape receptivity to meta-respect interventions, as these factors may moderate their perceived credibility and effectiveness. It is also important to explore alternative ways of conveying intergroup meta-respect that allow for evaluating the psychological impact of authentic intergroup communication or recognition experiences. Finally, future studies should assess the durability of these effects. For instance, how long do corrected meta-respect perceptions persist, and under what conditions might their impact be diminished or strengthened? This could include longitudinal and follow-up designs to track the persistence of these perceptions, as well as experimental approaches that expose participants to repeated or contextually reinforced respect-based messages. By deepening our understanding of how respect and meta-respect function in conflict settings, scholars, educators, and practitioners alike may identify new pathways toward fostering positive perceptions of the outgroup, encouraging interaction between the groups, and ultimately promoting mutual respect.

## Figures and Tables

**Table 1:** Frequencies of Israeli Jews’ Perceptions of Arabs’ Deservedness of Respect and Meta-Respect (Study 1).

Jewish Sample					
Think That Arabs Deserve Respect			Perceived Respect from Arabs (Meta-Respect)		
Deserve/not deserve respect	Frequency (percent)	Cumulative frequency (percent)	Deserve/not deserve respect	Frequency (percent)	Cumulative frequency (percent)
Strongly deserve	22.2	64.3	Strongly deserve	7.1	30.2
Deserve	28.2		Deserve	7.1	
Fairly deserve	13.9		Fairly deserve	15.9	
Fairly not deserve	9.9	35.7	Fairly not deserve	11.1	69.8
Not deserve	10.3		Not deserve	17.9	
Strongly not deserve	15.5		Strongly not deserve	40.9	

*Note.* Percentages are based on Jewish participants’ perceptions (*N* = 252).

**Table 2:** Frequencies of Israeli Arabs’ Perceptions of Jews’ Deservedness of Respect and Meta-Respect (Study 1).

Arab Sample					
Think That Jews Deserve Respect			Perceived Respect from Jews (Meta-Respect)		
Deserve/not deserve respect	Frequency (percent)	Cumulative frequency (percent)	Deserve/not deserve respect	Frequency (percent)	Cumulative frequency (percent)
Strongly deserve	41.2	83.9	Strongly deserve	11.6	46.2
Deserve	29.6		Deserve	13.1	
Fairly deserve	13.1		Fairly deserve	21.6	
Fairly not deserve	10.6	16.1	Fairly not deserve	16.6	53.8
Not deserve	3.0		Not deserve	21.6	
Strongly not deserve	2.5		Strongly not deserve	15.6	

*Note.* Percentages are based on Arab participants’ perceptions (*N* = 199).

**Table 3:** Variable Descriptive Statistics and Correlations in the Jewish Sample (N = 252, Study 1).

Measures	*M*	*SD*	1	2	3	4
1. Outgroup deserves respect	3.96	1.75	--			
2. Meta-respect	2.52	1.63	0.38 **	--		
3. Outgroup humanization	4.80	3.22	0.79 **	0.51 **	--	
4. Political orientation (+left)	2.15	0.88	0.39 **	0.32 **	0.41 **	--

Note. ** *p* < 0.01 (two-tailed).

**Table 4:** Variable Descriptive Statistics and Correlations in the Arab Sample (N = 199, Study 1).

Measures	*M*	*SD*	1	2	3
1. Outgroup deserves respect	4.88	1.27	--		
2. Meta-respect	3.30	1.59	0.28 **	--	
3. Outgroup humanization	6.11	2.54	0.59 **	0.48 **	--

Note. ** *p* < 0.01 (two-tailed). Political orientation was not included in the analyses because nearly half of the Arab participants either did not vote or did not report their political orientation.

**Table 5:** Frequencies of Israeli Jews’ Perceptions of Arabs’ Respect Toward Jews (Meta-Respect) (Study 2).

Jewish Sample			
Perceived Respect from Arabs (Meta-Respect): % of Participants Who Believe Arabs View Jews as Deserving/Not Deserving of Respect			Perceived Respect from Arabs (Meta-Respect): % of Arabs Who Believe Jews Deserve Respect (Average)
Deserve/not deserved respect	Frequency (percent)	Cumulative frequency (percent)	29.52%
Strongly deserve	1.8	20.6	
Deserve	5.5		
Fairly deserve	13.3		
Fairly not deserve	20.6	79.4	
Not deserve	23		
Strongly not deserve	35.8		

*Note.* Percentages are based on Jewish participants’ perceptions (*N* = 165).

**Table 6:** Frequencies of Israeli Arabs’ Perceptions of Jews’ Respect Toward Arabs (Meta-Respect) (Study 2).

Arab Sample			
Perceived Respect from Jews (Meta-Respect): % of Participants Who Believe Arabs View Arabs as Deserving/Not Deserving of Respect			Perceived Respect from Jews (Meta-Respect): % of Jews Who Believe Arabs Deserve Respect (Average)
Deserve/not deserve respect	Frequency (percent)	Cumulative frequency (percent)	46.14%
Strongly deserve	9.3	47.8	
Deserve	12.4		
Fairly deserve	26.1		
Fairly not deserve	19.9	52.2	
Not deserved	19.9		
Strongly not deserve	12.4		

*Note.* Percentages are based on Arab participants’ perceptions (*N* = 161).

**Table 7:** Means (SDs) and One-Way ANOVAs Comparing Three Conditions (Study 2 Among Jews).

DV	Control	Outgroup Respect	Meta-Respect Correction	F (2, 164)	*p*	η^2^	Post Hoc
Feeling respect	1.67 (1.02)	2.27 (1.36)	2.38 (1.24)	5.35	<0.01	0.06	* Exp1, ** Exp2 > Control
Hope	1.59 (1.03)	2.36 (1.40)	2.43 (1.26)	7.59	<0.001	0.08	** Exp1, ** Exp2 > Control
Positive light	1.93 (1.05)	2.38 (1.11)	2.38 (1.00)	3.25	0.041	0.04	^†^ Exp1, ^†^ Exp2 > Control
Outgroup humanization	4.57 (3.14)	5.40 (2.94)	4.68 (2.62)	1.31	0.272	0.01	--
Willingness to respect	3.12 (1.30)	3.62 (1.19)	3.23 (1.11)	2.57	0.079	0.03	--
Willingness to interact	3.07 (1.81)	3.30 (1.89)	2.98 (1.50)	0.51	0.597	0.006	--

Note. *N* = 165. Post hoc tests were Tukey HSD. Exp1 = Outgroup respect, Exp2 = Meta-respect correction. * *p* < 0.05, ** *p* < 0.01, ^†^ *p* = 0.073, 0.070.

**Table 8:** Means (SDs) and One-Way ANOVAs Comparing Three Conditions (Study 2 Among Arabs).

DV	Control	Outgroup Respect	Meta-Respect Correction	F (2, 160)	*p*	η^2^	Post Hoc
Feeling respect	2.32 (1.35)	3.47 (1.26)	3.15 (1.43)	10.40	<0.001	0.11	*** Exp1, ** Exp2 > Control
Hope	2.40 (1.30)	3.67 (1.10)	3.36 (1.27)	15.68	<0.001	0.16	*** Exp1, *** Exp2 > Control
Positive light	3.02 (1.12)	3.68 (.94)	3.52 (.93)	6.19	<0.01	0.07	** Exp1, * Exp2 > Control
Outgroup humanization	6.23 (2.58)	7.33 (2.19)	6.42 (2.24)	3.41	0.035	0.04	* Exp1 > Control
Willingness to respect	3.96 (1.04)	4.24 (.82)	4.08 (.87)	1.27	0.284	0.01	--
Willingness to interact	5.48 (1.52)	5.92 (1.30)	5.57 (1.41)	1.44	0.240	0.02	--

Note. *N* = 161. Post hoc tests were Tukey HSD. Exp1 = Outgroup respect, Exp2 = Meta-respect correction. * *p* < 0.05, ** *p* < 0.01, *** *p* < 0.001.

**Table 9:** Indirect Effects of Outgroup Respect and Meta-respect Correction on Intergroup Attitudes Through Feeling Respected, Controlling for Political Orientation (Study 2, Among Jews).

Condition	Dependent Variable	*b XM*	*b MY*	Indirect Effect
**Outgroup respect vs. Control (*n* = 109)**	Outgroup humanization	−0.30 **	0.68 **	b = −0.21, SE = 0.09, 95% CI = −0.40 to −0.03
				TE: b = −0.47, SE = 0.26, 95% CI = −1.00 to 0.06
	Willingness to respect the outgroup	−0.30 **	0.31 ***	b = −0.09, SE = 0.03, 95% CI = −0.17 to −0.02
				TE: b = −0.27, SE = 0.10, 95% CI = −0.48 to −0.06
	Willingness to interact with the outgroup	−0.30 **	0.47 ***	b = −0.14, SE = 0.06, 95% CI = −0.27 to −0.02
				TE: b = −0.16, SE = 0.15, 95% CI = −0.46 to 0.13
**Meta-respect correction** **vs. Control (*n* = 110)**	Outgroup humanization	−0.71 **	0.63 **	b = −0.44, SE = 0.19, 95% CI = −0.82 to −0.07
				TE: b = −0.20, SE = 0.51, 95% CI = −1.22 to 0.80
	Willingness to respect the outgroup	−0.71 **	0.27 **	b = −0.19, SE = 0.08, 95% CI = −0.35 to −0.03
				TE: b = −0.15, SE = 0.20, 95% CI = −0.56 to 0.25
	Willingness to interact with the outgroup	−0.71 **	0.29 *	b = −0.20, SE = 0.12, 95% CI = −0.43 to 0.03
				TE: b = 0.01, SE = 0.27, 95% CI = −0.53 to 0.56

Note. XM = Respect condition on feeling respected. MY = Feeling respected on dependent variable. Coefficients are unstandardized. TE = Total effect. * *p* < 0.05, ** *p* < 0.01, *** *p* < 0.001.

## Data Availability

The data of this article are available in OSF at: https://osf.io/4zejk/overview?view_only=79939bea7ecb4b778ee5fae9d892d6d8 (accessed on 24 October 2025).

## Supplementary Materials

The following supporting information can be downloaded at: https://www.mdpi.com/article/10.3390/bs15111474/s1, Table S1: Demographic of excluded participants based on failed attention check (Study 2).
