# Supplementary material for: Intergroup Meta-Respect Perceptions in a Context of Conflict
Source: Behav Sci (Basel). 2025 Oct 29;15(11):1474. doi: 10.3390/bs15111474 (PMC12649413; doi:10.3390/bs15111474)
Supplement: Supplementary file 1 [file behavsci-15-01474-s001.zip › behavsci-3876243-supplementary.pdf]

Intergroup Meta-Respect Perceptions in a Context of Conflict

Supplementary materials

Table S1: Demographic of excluded participants based on failed attention check (Study 2)

Jewish Sample

**Excluded participants (N=17)**		**Included participants (N=165)**	
Gender	53% men, 47% women	Gender	47% men, 53% women
Age	M= 30.82, SD=8.23	Age	M= 33.09, SD=9.12
Religiosity	41% secular, 41%, traditional, 6% religious, 12% ultra-orthodox	Religiosity	46% secular, 36% traditional, 10% religious, 8% ultra-orthodox
Condition	Condition 1 = 7<br>Condition 2 = 10	Condition	Condition 1 = 55<br>Condition 2 = 56

Arab Sample

**Excluded participants (N=28)**		**Included participants (N=161)**	
Gender	39% men, 61% women	Gender	40% men, 60% women
Age	M= 35.93, SD=8.28	Age	M= 34.18, SD=8.33
Religion	82% Muslims, 4% Christians, 14% Druze	Religion	70% Muslims, 12% Christians, 18% Druze
Religiosity	39% secular, 39%, traditional, 14% religious, 8% were not identified	Religiosity	35% secular, 44% traditional, 21% religious
Condition	Condition 1 = 14<br>Condition 2 = 14	Condition	Condition 1 = 55<br>Condition 2 = 53
